# Extensive alterations of blood metabolites in pediatric cerebral malaria

**DOI:** 10.1371/journal.pone.0175686

**Published:** 2017-04-20

**Authors:** Sanchit Gupta, Karl Seydel, Miguel A. Miranda-Roman, Catherine M. Feintuch, Alex Saidi, Ryung S. Kim, Gretchen L. Birbeck, Terrie Taylor, Johanna P. Daily

**Affiliations:** 1 Department of Medicine, Division of Infectious Disease, Albert Einstein College of Medicine, Bronx, New York, United States of America; 2 Blantyre Malaria Project, University of Malawi College of Medicine, Blantyre, Malawi; 3 Department of Osteopathic Medical Specialties, College of Osteopathic Medicine, Michigan State University, East Lansing, Michigan, United States of America; 4 Department of Microbiology and Immunology, Albert Einstein College of Medicine, Bronx, New York, United States of America; 5 Department of Epidemiology and Population Health, Albert Einstein College of Medicine, Bronx, New York, United States of America; 6 Department of Neurology, Epilepsy Division, University of Rochester, Rochester, New York, United States of America; National Research Council of Italy, ITALY

## Abstract

Cerebral malaria (CM) presents as an encephalopathy and is due to infection with *Plasmodium falciparum*. Patients are comatose, often with fever, recurrent seizures and this condition is associated with a high mortality rate. The etiology of the coma and seizures are poorly understood. Circulating small molecules and lipids have bioactive functions and alterations in their concentrations have been implicated in seizure disorders and other forms of encephalopathy. We carried out a comprehensive analysis of blood metabolites during CM to explore a biochemical basis of this encephalopathy. A paired metabolomics analysis was performed on the plasma samples of Malawian children (n = 11) during CM and at convalescence thirty days later, to identify differentially abundant molecules associated with CM. We also report plasma molecules associated with CM mortality (n = 4) compared to survival (n = 19). Plasma metabolites were identified through ultra high performance liquid chromatography/tandem mass spectrometry and gas chromatography/mass spectrometry to maximize compound detection and accuracy and then compared to a library for identification. We detected a total of 432 small molecules in the plasma and 247 metabolites were significantly differentially abundant between CM and convalescence (p < 0.05, FDR < 0.10). These represented global changes across many classes of molecules including lipids, amino acids and hemoglobin metabolites. We observed significant changes in molecules that could impact neurologic function during CM; these include increased levels of kynurenate and decreased indolepropionate, glutamate, arginine and glutamine. Moreover, 1-methylimidazoleacetate, kyurenate, arachidonic acid and dimethylarginine were associated with mortality (p < 0.05, fold change > 1.2). These results highlight the broad changes in blood chemistry during CM. We have identified metabolites that may impact central nervous system physiology and disease outcomes and can be further explored for their mechanistic roles into the pathophysiology of CM.

## Introduction

Despite improvements in clinical care and vector control, *Plasmodium falciparum* malaria remains a disease of global importance, with 214 million cases worldwide and 438,000 deaths, mostly among children in Africa [1]. Cerebral malaria (CM), a severe complication of *P*. *falciparum* infection is heralded by coma, and a high prevalence of recurrent seizures and status epilepticus [2]. The encephalopathy and seizures typically resolve within 2–3 days in conjunction with antimalarial treatment and supportive care, but 25% of pediatric CM survivors develop long term neurological sequelae including epilepsy [3]. The mechanisms of seizure and coma in CM remain poorly understood.

The rapid onset and resolution of the coma of CM are consistent with a metabolic cause. Metabolic changes in blood are common during CM and include hypoglycemia and hyperlactatemia, though a comprehensive analysis has not been reported [4]. Changes in blood composition reflect the physiology of a severe *P*. *falciparum* infection which is associated with high levels of inflammation, hemolysis and the presence of parasite derived metabolites. Local tissue hypoxia due to microvascular compromise from sequestration of infected red blood cells (iRBC) to vascular endothelial cells further impacts blood metabolite levels. Taken together, we hypothesized that significant changes in the blood metabolome occur during CM, and that these changes could impact normal brain functions. To examine the potential role of small molecules and lipids in the clinical findings of CM we carried out a comprehensive, unbiased plasma metabolomics association study in a cohort of Malawian children with CM during coma and during a convalescent state thirty days later.

## Materials and methods

### Study population

Study subjects were Malawian children aged 6 months to 12 years enrolled in the Blantyre Malaria Project (BMP), who presented with CM during the malaria transmission season January through June 2013 [5, 6]. Study subjects had a Blantyre Coma Score ≤ 2, with no other identified cause of coma such as hypoglycemia, bacterial meningitis or a post ictal state. Children who fulfilled the WHO case definition of CM and had evidence of iRBC brain microvasculature sequestration determined by the presence of retinopathy were studied [5]. Patients were excluded from the analysis if they were HIV positive, determined by two rapid tests: Uni-Gold (Trinity Biotech) and Determine (Inverness Medical). Upon enrollment, venous blood samples were collected in EDTA collection tubes, and plasma was separated by centrifugation and maintained at -80°C. Study subjects were treated with intravenous quinine (in accordance with Malawi government treatment guidelines at that time), provided supportive care during their hospitalization and were negative for parasites on blood smears, prior to discharge from the research ward. Study survivors presented for follow up one month later and underwent a physical exam, blood smear and a repeat venipuncture to obtain a convalescent plasma sample. Clinical and laboratory data were extracted from the study subjects’ record. The study was approved by The University of Malawi College of Medicine Research and Ethics Committee, and by the Institutional Review Boards of Michigan State University and Albert Einstein College of Medicine. Written informed consent was obtained from the accompanying parent or guardian of the study subjects.

### Metabolomic analysis

To identify blood metabolites, a non-targeted metabolomics analysis was carried out on plasma using ultra high performance liquid chromatography/tandem mass spectrometry in both positive and negative ion modes and gas chromatography/mass spectrometry to maximize compound detection and accuracy (Metabolon, Durham, NC). Metabolites were identified by comparing the experimental samples’ spectral signatures to a reference library dataset [7]. The data provide relative molecule abundance represented by ion counts. We then quantified and validated a subset of molecules that demonstrated significant differences using Absolute*IDQ*^TM^ (BIOCRATES Life Sciences AG, Innsbruck, Austria) on an UPLC-MS/MS platform (Acquity UPLC- Xevo TQ MS, Waters Corporation, Milford, MA, USA) using methods as described [8]. This assay also quantified phospholipids and sphingolipids. Metabolites with a covariance less than 20% are reported.

### Statistical analysis

The primary outcome was to identify plasma small molecules that were significantly differentially abundant during CM compared to the paired convalescent sample, and the secondary outcome was small molecules associated with mortality. Ion counts were generated for each metabolite and the metabolite was retained in the analysis if it was detected in 50% or more samples from either the CM or convalescent group. After this filter, minimum values across all groups were imputed for any remaining data below the detection threshold. Fold change for abundance was calculated relative to the CM sample as compared to the matched convalescent value, and in death relative to survival in the mortality analysis. Ion count values were log2-transformed and analyzed by paired t-test for the CM-convalescent comparison, and t-test with Welch approximation for the mortality analysis. The false discovery rate (FDR) was calculated for each metabolite for both comparisons. We defined significant metabolites as having a p < 0.05 and FDR < 0.10 comparing paired CM and convalescent samples. For the survival analysis we used a p < 0.05 without a FDR due to the low number of deaths. Comparisons of CM and convalescence plasma amino acid concentrations were performed using the Wilcoxon matched-pairs signed rank test. Analyses were carried out with R v3.1.1 (http://cran.r-project.org/), Stata v13.0 (StatCorp, College Station, TX) and Graph Prism (GraphPad Software, La Jolla, CA). GenePattern (http://www.broadinstitute.org/cancer/software/genepattern/) was used for hierarchical clustering analysis and heat map generation [9].

## Results

### Characteristics of the study cohort

During the 2013 transmission seasons, plasma samples were collected on admission from 43 pediatric patients with CM and from 31 survivors on day 30 post-admission. Eleven patients without retinopathy were excluded. Twenty-three CM patient plasma samples were randomly selected for metabolomics analysis (Table 1). The demographics, laboratory data and outcomes of this subset were not statistically different from the entire cohort (data not shown). The twenty-three subjects manifested fever, coma, tachycardia, anemia and thrombocytopenia reflective of CM. Four patients (17%) died, and at the time of discharge, 17% (4) of the analyzed subjects sustained neurologic sequelae such as deficits in vision, hearing, or motor function.

**Table 1:** Associated clinical and laboratory features of Malawian children with cerebral malaria that underwent plasma metabolic profiling (n = 23).

Feature	Median (IQR)
**Demographic and clinical**	
Age (months)	45 (28–74)
Sex (% female)	57
Weight (kg)	11.7 (8.9–17.0)
Temperature (°C)	38.1 (37.5–39.0)
Systolic blood pressure (mmHg)	94 (86–101)
Pulse (bpm)	148 (128–164)
Respiratory rate (breaths/minute)	44 (36–54)
Coma resolution time (hrs)	60 (38–92)
Parasite clearance time (hrs)	36 (30–48)
Fever clearance time (hrs)	32 (18–72)
Neurologic sequelae (%)	17
Death (%)	17
**Laboratory**	
Hematocrit (%)	21 (18–28)
Parasitemia (x 10^3^/µL)	67 (5–345)
WBC (x10^3^/µL)	9.0 (5.9–13.5)
Platelets (x10^3^/µL)	59 (30–97)
Glucose (mg/dL)	96.4 (77.5–113.5)
Lactate (mmol/L)	6.0 (3.1–9.1)

IQR: Interquartile range.

To identify the blood metabolite profiles during CM compared to convalescence, we randomly selected eleven paired plasma samples. During the convalescent study visit, all eleven subjects were afebrile, awake and overall clinically well. The median convalescent hematocrit of 35% was significantly increased from the enrollment hematocrit of 23% (p = 0.008). Patient number 5 presented to the convalescent visit with a two-day history of fever. At the time of the exam he was afebrile, fully conscious, with a non-focal exam, however he had a 4+ positive (semi-quantitative measurement, 1+ to 5+) peripheral blood parasitemia. He was treated with an oral antimalarial. All of the other patients were blood smear negative. Patient 6 reported one week of diarrhea and was afebrile with a non-focal exam.

### Blood metabolite differences during CM compared to paired convalescence samples

**Fig 1:**
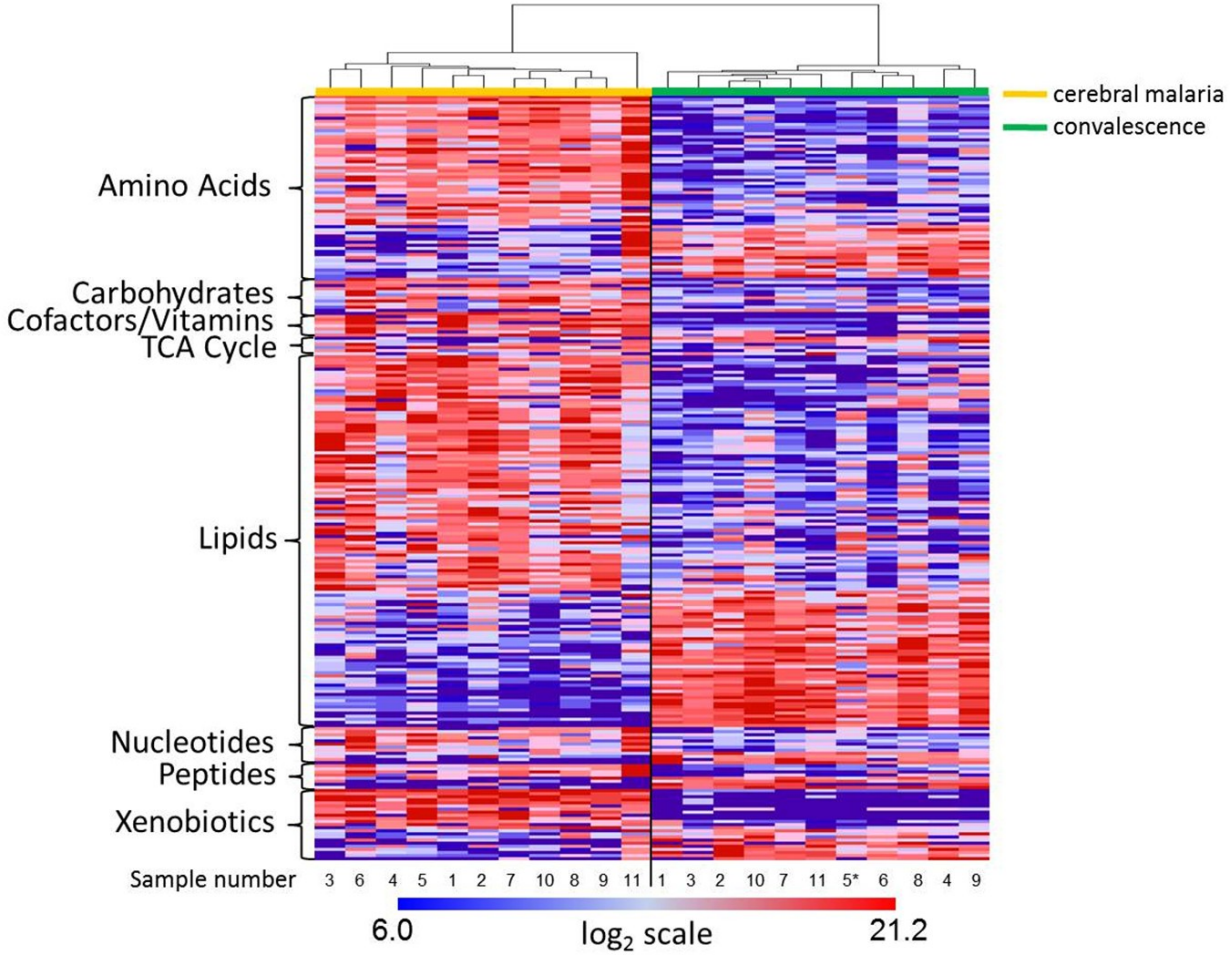
Hierarchical clustering of differentially abundant plasma metabolites during CM and convalescence in a paired analysis of eleven Malawian children. A heatmap of a heirarchical clustering of 247 differentially abundant (p < 0.05, FDR < 0.10, paired t test) plasma metabolites between CM and convalescence (one month later). Unsupervised hierchical clustering segregates the samples by clinical state. The asterisk notes one child with mild malaria diagnosed during the convalescent visit.

We detected a total of 432 metabolites of which 393 remained after filtering out infrequently detected molecules in the eleven paired samples. Of the 393 metabolites, 247 were statistically significantly differentially abundant (p < 0.05, FDR < 0.10) between CM and convalescence. These differentially abundant molecules represented diverse chemical classes, including 59 amino acids, 11 carbohydrates, 10 cofactors and vitamins, 4 tricarboxylic cycle acid (TCA) cycle intermediates, 120 lipids, 12 nucleotides, 8 peptides, and 23 xenobiotics (S1 Table). Unsupervised hierarchical clustering of these 247 metabolites completely segregated the samples by CM and convalescence (Fig 1). Notably, the metabolome derived from the convalescent sample (patient 5) who had mild malaria clustered with the convalescent samples.

**Fig 2:**
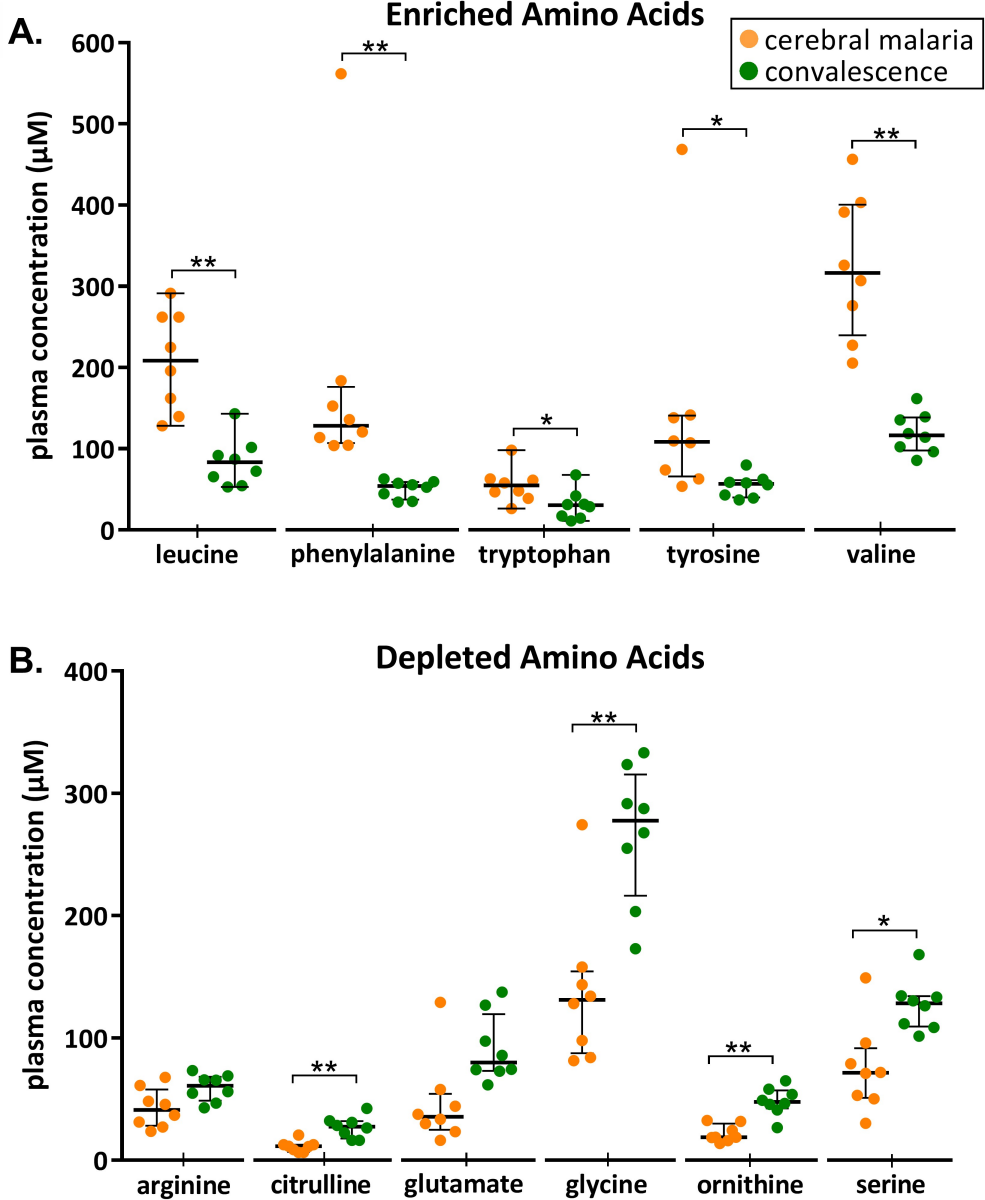
Plasma amino acid concentrations during CM compared to convalescence. Selected plasma amino acid concentrations during CM and at convalescence (one month later) in eleven children. (A) Enriched amino acids during CM. (B) Depleted amino acids during CM. Scatter plots show median and interquartile ranges. A Wilcoxon matched-pairs signed rank test was performed. * represents P<0.05; ** represents P<0.01.

Amino acids and their metabolites were both enriched and depleted during CM. Alpha-ketobutyrate and 2-hydroxybutyrate, degradation products of threonine and methionine metabolism, were highly enriched during CM (S1 Table). We quantified and validated the increase in a subset of amino acids during CM, including branched chained amino acids leucine (211 µM vs 80 µM, p = 0.008) and valine (317 µM vs 117 µM, p = 0.008) (Fig 2). Phenylalanine was also increased during CM (128 µM vs 54 µM, p = 0.008), as was tryptophan (53 µM vs 30 µM, p = 0.016). Phenylalanine metabolites 3-(4-hydroxyphenyl) lactate, phenyllactate and N-acetylphenylalanine were also enriched during CM, along with tryptophan metabolites kynurenine (6.4 µM vs 1.7 µM, p = 0.008), kynurenate and indolelactate (S1 Table).

During CM, we found a depletion in amino acids including arginine (41 µM vs 61 µM p = 0.109), glutamate (36 µM vs 80 µM p = 0.078) and glycine (131 µM vs 278 µM p = 0.008) (Fig 2). Citrulline (11 µM vs 27 µM p = 0.008) and ornithine (19 µM vs 48 µM, p = 0.008), metabolic precursors for arginine, the principle substrate for nitric oxide production, were also depleted during CM. Indolepropionate, a tryptophan derivative that acts as a neuroprotective free-radical scavenger, demonstrated a large reduction during CM (S1 Table) [10].

The study subjects were anemic during CM, with a median hematocrit of 21%. Red cell lysis was reflected with a significant enrichment of heme metabolites during CM including elevated biliverdin, bilirubin, L-urobilin, and I-urobilinogen (S1 Table).

**Fig 3:**
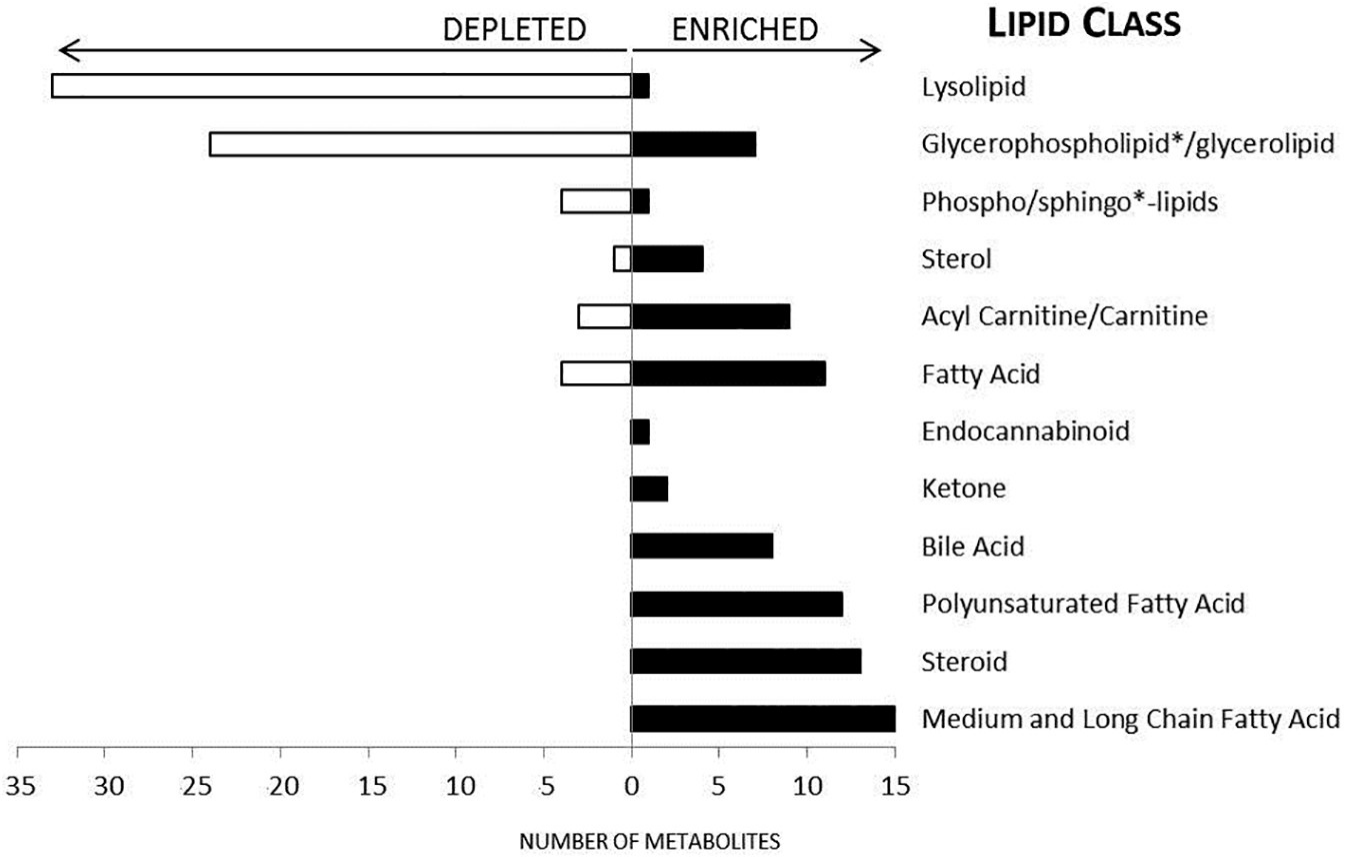
Enrichment in fatty acids, sterols, bile acids and other lipids during CM compared to convalescence. A bar graph of the number of differentially abundant lipids (paired t test, p-value < 0.05, FDR < 0.10) by lipid class during CM compared to convalescence (n = 11 paired samples). We found marked reduction in the number of phospholipids and lysolipids during infection and enrichment in fatty acids. * denotes data obtained from a quantitative metabolomic method (n = 8 paired samples).

Notably there was a generalized enrichment of fatty acids during CM, including 12 polyunsaturated fatty acids and 15 medium and long chain fatty acids with concomitant reduction in lysolipids (Fig 3, S1 Table). Arachidonic acid, which is mobilized from membrane lipids by phospholipase A2 (PLA2) and is a precursor for a wide variety of inflammatory mediators such as eicosanoids, was increased during CM compared to convalescence. To further explore and quantify the decrease in complex lipids, we carried out a quantitative analysis in 8 of the 11 paired samples and found a significant depletion of 22 lysolipids and enrichment of 5 species of phosphatidylcholine during CM (p < 0.05, FDR < 0.10) (Fig 3, S2 Table).

During CM, bile acids were broadly enriched and alterations in relative contribution of each bile acid to the total bile acid pool was detected (S3 Table) [11]. Four gamma-glutamyl moieties and thirteen steroids, including cortisone, cortisol and pregnenolone sulfate, were elevated during CM (S1 Table). Quinine and acetaminophen were detected during CM secondary to malaria treatment.

### Plasma molecules associated with mortality

To identify metabolites associated with mortality, we compared the CM metabolome dataset between fatal cases (n = 4) and survivors (n = 19). 363 metabolites were assessed after filtering, and 19 were significantly associated with death (p<0.05), including 1-methylimidazoleacetate, kyurenate, arachidonic acid and dimethylarginine (p < 0.05, fold change > 1.2, Table 2).

**Table 2:** Blood metabolites associated with mortality in CM (n = 23).

Molecule	Pathway	P value	Fold change
5-hydroxyhexanoate	Fatty Acid, Monohydroxy	4.7E-02	2.17
palmitoyl ethanolamide	Endocannabinoid	2.6E-02	1.86
docosadienoate (22:2n6)	Polyunsaturated Fatty Acid (n3 and n6)	2.8E-02	1.84
kynurenate	Tryptophan Metabolism	5.3E-03	1.78
1-methylimidazoleacetate	Histidine Metabolism	3.8E-03	1.51
arginine	Urea cycle; Arginine and Proline Metabolism	1.1E-02	1.51
N-acetylthreonine	Glycine, Serine and Threonine Metabolism	2.3E-02	1.46
17-methylstearate	Fatty Acid, Branched	1.2E-02	1.42
gluconate	Food Component/Plant	3.2E-02	1.34
acisoga	Polyamine Metabolism	3.6E-02	1.32
arachidonic acid (20:4n6)	Polyunsaturated Fatty Acid (n3 and n6)	8.0E-03	1.25
dimethylarginine (SDMA + ADMA)	Urea cycle; Arginine and Proline Metabolism	4.7E-02	1.20
arabinose	Pentose Metabolism	2.4E-02	1.18
uridine	Pyrimidine Metabolism, Uracil containing	1.7E-02	1.14
urea	Urea cycle; Arginine and Proline Metabolism	4.5E-02	1.13
valine	Leucine, Isoleucine and Valine Metabolism	2.8E-02	0.73
3-phenylpropionate (hydrocinnamate)	Phenylalanine and Tyrosine Metabolism	4.0E-05	0.30
taurolithocholate 3-sulfate	Secondary Bile Acid Metabolism	1.8E-02	0.22
1,2-propanediol	Chemical	4.4E-02	0.10

Plasma metabolites collected during CM were compared between 4 non-survivors and 19 survivors. Fold change represents the ratio of non survivors to survivors. Ion counts were log2-transformed and analyzed for significance by t-test with Welch approximation.

## Discussion

CM is a severe systemic disease associated with an encephalopathy, seizures and mortality. To examine a potential role of small molecules in the pathophysiology of pediatric CM we characterized the blood metabolomes during CM compared to convalescence. We identified greater than two hundred small molecules and lipids that were significantly altered during pediatric CM reflecting global blood chemistry dysregulation. Both parasite and host metabolism likely contributes to these changes during CM. A large sequestered biomass of parasites utilize host nutrients including carbons, lipids, purines and amino acids and these processes are accompanied by a catabolic inflammatory host response to alter the blood chemical landscape [12]. Some of the depleted molecules have important neuroprotective roles, such as indolepriopionate and the depletion of other molecules such as glutamate can impair immunologic function. More broadly, we noted a marked depletion of complex phospholipids and an enrichment in fatty acids representing an increase in energetic needs and evidence of liver dysfunction suggested by increases in bile acids. These data provide new insights into the pathophysiology and potential biochemical etiologies of the neurological manifestations and mortality of CM.

### Neurologic implications

Seizures commonly occur during CM and the development of epilepsy post CM is prevalent. In other conditions, metabolic causes of seizures are associated with abnormal levels of amino acids, purines, carbohydrates, acylcarnitines and urea cycle defects [13]. We found changes in small molecules levels during CM that have been implicated in seizure and/or encephalopathies, including increases in kynurenate and the branch chain amino acids leucine and valine [14]. We also noted a modest elevation in phenylalanine (2.6 fold increase), which has been reported in children and adults with CM and other forms of severe malaria compared to controls [15]; however, the blood concentrations did not approach those seen in phenylketonuria, which results in seizures and other neurological symptoms [16]. Oxidative stress may contribute to the development of epilepsy, as compounds with antioxidant properties prevent seizure induced pathology [17]. A number anti-oxidants were depleted during CM including indolepriopionate a neuroprotective free-radical scavenger which has been shown to provide a neuro protective effect in an animal model of ischemia along with the glutathione precursors, glutamate, and glycine [18]. Thus a reduction in molecules that combat oxidative stress may contribute to the high prevalence of seizures and post CM epilepsy [10]. Glutamate plasma levels were lower during CM compared to convalescence. This amino acid provides cellular energy and low levels can impair white blood cell function [19]. Treatment with glutamate could potentially improve outcomes as supplemental glutamate increased survival in the animal model of cerebral malaria through modulation of CD8+ effector T cell function [20].

### Hemoglobin metabolism and liver disease

We observed elevated heme degradation products including bilirubin, biliverdin and urobilinogen during CM. Heme is metabolized to biliverdin, then to bilirubin which is transported to the gastrointestinal tract and further metabolized by gut microbiota to urobilinogen. Elevations in heme metabolites can reflect the increase in heme metabolism due the hemolysis of malaria infection and/or liver injury [21]. The enrichment of gamma-glutamyl amino acids may indicate increased activity of gamma-glutamyl transferase, which is associated with liver disease [22]. High levels of bilirubin can cause neurotoxicity (kernicterus), though the impact of its downstream metabolites on brain function is unknown. Bile salts, which facilitate hepatobiliary secretion of lipids, and act as signaling molecules in metabolism and immune responses, were dysregulated during CM, and may also reflect liver disease [23]. Autopsy studies of pediatric CM have identified extensive Kupffer cell hyperplasia with accumulation of malaria pigment and mild portal and lobular lymphocytic infiltration, however there are only minimal signs of cellular necrosis or cholestasis [24]. Perhaps this unique pathology of the biliary system is sufficient to alter plasma bile salt concentrations. Individual bile salts have distinctive bioactive properties and can impact the blood brain barrier and neurologic function [25–27].

More broadly, we found a high level of ketone bodies, and an enrichment of fatty acids, with a decrease in lysolipids suggesting a shift to fatty acid metabolism, likely due to increased energetic needs [28]. High levels of catecholamines and pro-inflammatory cytokines during severe malaria may drive the enhanced lipolysis and *de novo* synthesis of fatty acids to be used for oxidative metabolism [29]. Liver stage parasites require phosphatidylcholine for growth and this may also contribute to decreased blood levels [30]. A study in the murine model of malaria found that blood lipoprotein profiles segregate animals that go on to cerebral as compare to non-cerebral malaria, at a time point prior to onset of neurologic symptoms, suggesting that lipid alterations may also be playing a role in neurologic function [31].

The reductions in plasma glycine, cysteine, serine, threonine and glutamine during CM could be secondary to parasite uptake and/or host utilization [32]. Arginine was low during CM, and its depletion plays an important role in the vasculopathy of CM [33]. Endothelial dysfunction may be further compounded by high blood levels of dimethylarginine, which can inhibit NO synthesis and was associated with death in our study [34].

### Biomarkers of mortality

We identified common changes in blood chemistry during CM and those reported in sepsis, which is also associated with inflammation and heightened mortality. Sepsis is also associated with an increase in 2-hydroxybutyrate, phenylalanine and decrease in glutamine, 1-arachidonoylglycerophosphocholine and 1-linoleoylglyerophosphocholine [35, 36]. Furthermore, elevated levels of kynurenate, 1-methylimidazoleacetate and dimethylarginine are associated with mortality in both conditions [37–39]. 1-methylimidazoleacetate is a histamine metabolite and histamine elevations have been previously reported during CM [40]. Histamine has been implicated in murine models of CM, with histamine receptor inhibition providing a protective effect [41]. A sepsis study and our data both found metabolites of PLA2 to be associated with death (1-arachidonoyl-GPE (20:4), 1-arachidonoyl-GPC (20:4) in sepsis, and arachidonic acid in CM) [37]. Despite the many shared clinical and biochemistry features between CM and sepsis, the neurological symptoms are unique to CM and thus the study of small molecules specifically associated with CM could provide mechanistic insights into CM associated seizures and coma.

One limitation of this study is the small sample size associated with death, and larger prospective studies are needed. Furthermore, our metabolomics method does not capture all molecules. Parasites can generate unique metabolites and thus additional extraction and analytic methods using unsupervised methods would be needed [42]. Understanding the impact of alterations of the metabolome in asymptomatic or mild malaria would require additional comparative analysis of these clinical phenotypes. Whether changes in single molecule or a combination of molecules in the setting of fever lowers the seizure threshold and/or contributes to the encephalopathy of CM is unknown. Longitudinal studies of children with CM with frequent blood sampling and small molecule correlations to mental status and EEG monitoring are underway to parse which molecules may be playing a role in the neurological manifestations of CM.

## Conclusions

Collectively, these data represent the first wide-scale blood metabolomics analysis during pediatric CM. Previous studies of blood chemistry during malaria infection malaria have been limited, with most using *in vitro* or animal models of disease, and none examining pediatric CM plasma. We see evidence of global changes across metabolite classes, many of which play important roles in human physiology. We found alterations in fatty acid metabolism and lipid distributions reflecting changes also described in sepsis and critical illness. Alterations in kynurenate, glutathione precursors, and glutamine may be involved in mediating encephalopathy and/or altering seizure thresholds. Further clinical and mechanistic studies are needed to understand the role of small molecules on neurologic function and mortality in CM to improve outcomes.

## Supporting information

S1 Table List of blood metabolite differences during CM compared to convalescence in a paired analysis. The 247 plasma small molecules that demonstrated statistically significantly differentially abundant ion counts (p < 0.05, FDR < 0.10) between CM and convalescence in eleven paired plasma samples are presented.
(XLSX)

S2 Table Quantification of blood lipids and other metabolites during CM compared to convalescence samples in a paired analysis. To quantify the changes in lipids and other selected metabolites, we carried out a quantitative analysis using Absolute*IDQ*^TM^ (BIOCRATES Life Sciences AG, Innsbruck, Austria) on an UPLC-MS/MS platform (Acquity UPLC- Xevo TQ MS, Waters Corporation, Milford, MA, USA) in eight pairs. Plasma concentrations, P values, FDR and fold changes are reported between CM and convalescence for all metabolites measured.
(XLSX)

S3 Table Differences in blood bile acids during CM compared to convalescence. Differences in ion counts, P values, FDR and fold changes are reported for bile acids in 11 paired plasma samples during CM and convalescence thirty days later.
(DOCX)
