# Supplementary material for: Extensive alterations of blood metabolites in pediatric cerebral malaria
Source: PLoS One. 2017 Apr 20;12(4):e0175686. doi: 10.1371/journal.pone.0175686 (PMC5398544; doi:10.1371/journal.pone.0175686)
Supplement: S3 Table — Differences in ion counts, P values, FDR and fold changes are reported for bile acids in 11 paired plasma samples during CM and convalescence thirty days later. (DOCX) [file pone.0175686.s003.docx]

**Supplemental Table 3. Elevated plasma bile acids during CM compared to convalescence**

| **Bile Acid (n=11 pairs)** | **P value** | **FDR** | **Fold Change** |
| --- | --- | --- | --- |
| **Primary Bile Acid** |  |  |  |
| taurochenodeoxycholate | 2.2E-03 | 5.8E-03 | 5.5 |
| taurocholate | 1.6E-03 | 4.6E-03 | 5.5 |
| **Secondary Bile Acid** |  |  |  |
| Glycocholenate sulfate* | 3.1E-02 | 5.2E-02 | 1.9 |
| Glycolithocholate sulfate* | 1.7E-02 | 3.1E-02 | 2.1 |
| Taurocholenate sulfate* | 1.0E-05 | 9.3E-05 | 8.4 |
| Taurodeoxycholate | 3.6E-03 | 8.7E-03 | 9.1 |
| Taurolithocholate 3-sulfate | 1.9E-04 | 8.6E-04 | 13.9 |
| Tauroursodeoxycholate | 4.0E-02 | 6.5E-02 | 7.5 |

Ion counts were log2-transformed and analyzed by paired t-test for the CM-convalescent comparison. False discovery rate (FDR) was calculated for each metabolite. * indicates compounds that are not based on a standard.
